# Suicide Rate, Depression and the Human Development Index: An Ecological Study From Mexico

**DOI:** 10.3389/fpubh.2020.561966

**Published:** 2020-11-17

**Authors:** Héctor Cabello-Rangel, María Elena Márquez-Caraveo, Lina Díaz-Castro

**Affiliations:** ^1^Diagnosis Auxiliary Division, Psychiatric Hospital “Fray Bernardino Álvarez”, México City, Mexico; ^2^Research Division, Children Psychiatric Hospital “Dr. Juan N. Navarro”, Mexico City, Mexico; ^3^Direction of Epidemiological and Psychosocial Research, National Institute of Psychiatry “Ramón de la Fuente Muñiz”, México City, Mexico

**Keywords:** human development index, suicide, depression, young, social determinants

## Abstract

**Objective:** To assess the contribution of depression, the human development index (HDI) including the health, education and income indexes as well as the households structure to the suicide rate in Mexican population from 15 to 49 years old.

**Methods:** An ecological cross-sectional study was carried out in people between 15 and 49 years old. The health index (HI), education index (EI), income index (II), and HDI were constructed. The suicide rate, educational level, per capita income, poverty, and rate of households were collected from official databases. Pearson's correlation coefficient (r) was used to determine the strength between the suicide rate and the per capita income, unemployment, poverty, HI, EI, II, HDI, non-family household, and depression incidence rate. A multiple linear regression model was used to know the association between suicide rates and HDI.

**Results:** The suicide rate was 8.76/100,000 inhabitants. The HDI of the 32 Mexican states were low 16%, middle 41%, high 22%, and extremely high 13%. A direct and positive intensity relationship between suicide rate and non-family households, was found (*r* = 0.352; *p* < 0.001); on the other hand, the suicide rate is significantly and negatively related to family households with Pearson (*r* = −0.350; *p* < 0.001).

**Conclusion:** The states of the Mexican Republic with the highest prevalence of non-family households had a positive association with the suicide rate. Based on the result of this study, it is possible to assume that, as the HDI increases, there is a greater possibility of living alone and having suicidal behavior.

## Introduction

Suicide is a public health problem that has increased in recent years, the World Health Organization (WHO) reports 804,000 suicides annually and it is the second leading cause of violent death in the population aged 15–29 years (1). In Mexico, deaths by suicide have increased in recent decades, and they are a significant cause of disability, it is estimated that the Disability Adjusted Life Years (DALYs) for suicide are 304.64/100,000 inhabitants according to the Institute for Health Metrics and Evaluation (IHME) (2).

Suicide, as other non-communicable diseases, is a multifactorial and multilevel phenomenon; caused by individual factors such as mental disorders (depression, psychosis, and substance abuse), and social factors such as stress, social exclusion, unemployment, working conditions, social support and violence, factors that are considered as social determinants that influence people's health (3).

Because economic conditions affect people's health and human development, preserving health is an investment in human capital, becoming a determinant of development and a way to overcome social inequalities. Under this perspective, the United Nations Development Program (UNDP), recognizes health as a part of human development and defines it as: “the expression of the freedom of people to live a long, healthy and creative life; pursue objectives that they themselves consider valuable; and actively participate in the sustainable and equitable development of the planet they share” (4).

The theory of human development assumes that better economic conditions do not ensure higher levels of health, therefore it is considered that the increase in Gross Domestic Product (GDP) per capita, is not a good measure for the impact on health; in contrast, the Human Development Index (HDI) could be a good indicator, due to it including three essential aspects of human life: (1) the possibility of having a long and healthy life; (2) the possibility of acquiring knowledge, and (3) the opportunity to have resources for a decent life (5).

In this sense, countries with low HDI have higher death rates from infectious diseases as opposed to countries with high HDI. In the latter case, the rate of non-communicable diseases (cancer, sedentary lifestyle, use of psychoactive substances) is higher than the observed in countries with medium or low HDI (6). Although high HDI countries have a higher life expectancy, educational level, and income, they also suffer from higher suicide rates for both men and women. In addition, in these countries, another variable such as unemployment is associated with suicide (7); and psychiatry disorders are reported to be present in 80 to 90% of suicide deaths (8). According to this meta-analysis this association is less undimmed in low-middle income countries (LMICs) but in spite of the heterogeneity of the studies, 58% of the deaths by suicide were associated with psychiatric disorders, saliently, mood disorders.

It is essential to mention that suicide in Mexico (composed of 32 states), is the second cause of death in those under 25 years of age (2), in spite of the fact that worldwide prevalence of suicide has decreased by 26%, it has increased 17.1% in Mexico (9). Interestingly, most of the studies in this regard have focused on the description of suicidal behavior and demographic related variables, for example, a descriptive analysis of the sociodemographic characteristics of people who committed suicide showed that of 924 suicides, 82.2% were men, the average age was 40 years, 75% were informal employment, and only 34% were married (10–13), but no study reported the association of suicide with indicators of human development or household structure.

In this context, the aim of the present study is to analyze the relationship between suicide rate and income, educational, health indexes, household's structure and depression in Mexican population from 15 to 49 years old.

## Materials and Methods

### Study Design

Exploratory ecological cross-sectional study, the unit of analysis is suicide rate (dependent variable) in the Mexican population between 15 and 49 years old, related to variables of income unemployment, educational level, poverty, households structure, human development index and depression incidence rate (independent variables).

### Measures

The suicide death rate concerning the total of violent deaths, the per capita income, educational level, and unemployment were obtained from the basic indicators and economic information from the databases of the National Institute of Statistics and Geography for year 2017 (Instituto Nacional de Estadística y Geografia, INEGI), the data corresponded to the last year available (14, 15). Regarding the type of households, INEGI defines households as family and non-family; family households can be nuclear, extended or compound, and non-family households include co-residents and single-person household (16). For study purposes, we included family household and non-family households. The poverty rate was obtained from the database of the National Council for the Evaluation of Social Development Policy for year 2018 (Consejo Nacional de Evaluación de la Política de Desarrollo Social, CONEVAL) (17), and the life expectancy from the database of the National Population Council (Consejo Nacional de Población, CONAPO) (2018) (18).

The depression rate was obtained from the morbidity reported by the General Directorate of Epidemiology of the Ministry of Health in Mexico in the year 2018 (Ministry of Health, Secretaría de Salud) (19), the data corresponds to the most recent national databases available in Mexico.

First of all, for each state of the Mexican Republic, three indexes were constructed, the health index (HI), the education index (EI) and the income index (II), based on the UNDP methodology for the construction of the Human Development Index (HDI) (20).

$$\begin{array}{l} Index=\frac{Value\;for\;the\;state\;of\;the\;Mexican\;Republic-Maximun\;value}{Maximun\;value-Minimum\;value} \end{array}$$

Subsequently, the HDI for each state was calculated with the formula:

$$\begin{array}{l} HDI=HI^{\frac{1}{3}}*{EI}^{\frac{1}{3}}*II^{\frac{1}{3}}\; \end{array}$$

HDI has several main components that include life expectancy at birth, mean years of formal education and expected years of formal education (21) and gross national income (GNI) per capita (2018 US dollars). According to classification of UNDP of HDI the countries are classification as follows 0.667–0.720, Low HDI; 0.723–0.742, Medium HDI; 0.745–0.760, High HDI; and 0.760–0.830, Very high HDI; the same manner the states of the Mexican Republic were classified.

### Data Analysis

For the analysis of the study variables, the Software STATA version 14 was used. Means and standard deviation of all the variables were calculated. Taking the national mean suicide rate of 6/100,000 inhabitants as a point of reference, two groups were obtained: group 1 with high suicide rates (>6/100,000 inhabitants), and group 2 with low suicide rates (≤ 6/100,000 inhabitants), to contrast the study variables in the two subsamples, a *t*-test was applied.

Pearson's correlation coefficient (r) was used to determine the strength and direction of a linear equation between the suicide rate and the indicator variables income per capita, unemployment, poverty, HI, EI, II, HDI, non-family households and family households, and depression incidence rate.

A multiple linear regression model (MLR) was used to predict probability of suicide rate occurrence. The stepwise entry-removal of the various explanatory variables (observed in the Pearson's correlation) allowed identifying those that had statistically significant influence on the probability of determining suicide rate. For this, different specifications of regression models were compared and the best goodness-of-fit- was chosen.

## Results

The descriptive analysis showed that 10% of violent deaths in Mexico correspond to suicides; young people between 15 and 49 years old showed an average rate of 8.76 (2.8 SD) suicides per 100,000 inhabitants. The average education is basic, with 9 years (0.8 SD) of educational level. Forty percentage of the population suffers poverty. Eighty-eight percentage of the households are familiar, that is, households with more than one person, the rest of the households are considered non-family, this type of households are mainly located in states of the country such as Mexico City, Baja California and Quintana Roo (Table 1).

**Table 1:** Descriptive statistics of suicide rate, depression, HDI and social variables at the country and state level.

	SR^*^	I (MXN)^*^	Ue^*^	EL^*^	PP (%)^**^	FH (%)^*^	NonFH^*^ (%)	**GINI**	**HI**	**EI**	**InI**	**HDI**	**DR+**
**Estados Unidos Mexicanos**	**Min-Max; Mean; SD**	**Min-Max; Mean; SD**	**Min-Max; Mean; SD**	**Min-Max; Mean; SD**	**Min-Max; Mean; SD**	**Min-Max; Mean; SD**	**Min-Max; Mean; SD**	**Min-Max; Mean; SD**	**Min-Max; Mean; SD**	**Min-Max; Mean; SD**	**Min-Max; Mean; SD**	**Min-Max; Mean; SD**	**Min-Max; Mean; SD**
	4.7–15.3; 8.75; 2.83	26,510–79,085; 49,130; 12,161	1.7–7.1; 3.38; 1.01	7.3–11.1; 9.14; 0.82	14.5–76.4; 39.94; 15.11	80.2–92.8; 88.41; 2.62	7.1–18.4; 11.08; 2.36	0.42–0.54; 0.48; 0.03	0.81–0.87; 0.85; 0.01	0.62–0.88; 0.69; 0.05	0.61–0.77; 0.69; 0.04	0.68–0.84; 0.741; 0.03	32.59–268.82; 111.03; 64.10
Aguascalientes	9.93	59,346	3.6	9.7	26.2	89.3	10.4	0.51	0.86	0.72	0.73	0.76	225.13
Baja California	6.97	59,178	2.9	9.8	23.3	85.2	14.1	0.51	0.86	0.78	0.73	0.77	107.68
Baja California Sur	8.73	68,778	4.2	9.9	18.1	85.5	13.1		0.86	0.69	0.75	0.77	133.85
Campeche	15.09	47,700	3.8	9.1	46.2	90.7	9.0	0.51	0.84	0.69	0.70	0.74	141.60
Chiapas	9.1	26,510	2.7	7.3	76.4	91.3	8.4	0.54	0.83	0.62	0.61	0.68	61.23
Chihuahua	10.55	54,030	2.7	9.5	26.3	86.4	12.9	0.47	0.85	0.70	0.72	0.75	181.33
Ciudad de México	9.95	79,085	4.6	11.1	30.6	83.4	15.3	0.52	0.87	0.88	0.77	0.84	32.59
Coahuila	7.99	55,925	4.4	9.9	22.5	89.2	10.4	0.48	0.85	0.72	0.72	0.76	268.82
Colima	8.22	52,766	3.6	9.5	30.9	87.6	11.7	0.42	0.85	0.70	0.71	0.75	203.51
Durango	7.4	43,648	3.8	9.1	37.3	87.6	12.3	0.47	0.84	0.70	0.68	0.74	250.26
Guanajuato	9.91	46,142	3.5	8.4	43.4	91.0	8.7	0.43	0.84	0.65	0.69	0.73	46.25
Guerrero	5.64	29,334	1.7	7.8	66.5	91.0	8.7	0.51	0.81	0.63	0.62	0.68	53.06
Hidalgo	5.94	38,783	2.9	8.7	43.8	88.6	11.1	0.47	0.84	0.69	0.67	0.73	79.94
Jalisco	10.44	60,541	2.8	9.2	28.4	87.9	11.4	0.46	0.85	0.69	0.73	0.76	133.83
México	6.71	48,013	4.1	9.5	42.7	91.4	8.5	0.47	0.85	0.69	0.70	0.74	57.34
Michoacán	8.28	42,653	2.6	7.9	46.0	88.0	11.6	0.49	0.84	0.64	0.68	0.71	94.97
Morelos	7.47	42,973	2.1	9.3	50.8	88.5	11.1	0.42	0.85	0.71	0.68	0.74	121.90
Nayarit	6.94	48,148	3.4	9.2	34.8	87.2	11.9	0.49	0.85	0.68	0.70	0.74	134.02
Nuevo Leon	7.24	68,959	3.8	10.3	14.5	90.0	9.5	0.50	0.86	0.74	0.75	0.78	73.83
Oaxaca	6.51	31,592	2.2	7.5	66.4	91.7	8.0	0.51	0.83	0.62	0.63	0.69	37.99
Puebla	7.12	38,975	2.7	8.5	58.9	90.0	9.7	0.48	0.84	0.68	0.67	0.73	41.16
Querétaro	7.11	61,339	4.3	9.6	27.6	89.4	10.0	0.49	0.85	0.73	0.73	0.77	43.91
Quintana Roo	14.73	56,711	3.2	9.6	27.6	80.2	18.4	0.48	0.85	0.68	0.72	0.75	75.75
San Luis Potosí	9.29	46,497	2.3	8.8	43.4	89.7	10.0	0.51	0.84	0.67	0.69	0.73	128.52
Sinaloa	4.67	55,474	3.6	9.6	30.9	88.2	11.4	0.47	0.84	0.72	0.72	0.76	184.48
Sonora	11.4	59,883	3.7	10	28.2	84.7	14.9	0.48	0.85	0.72	0.73	0.77	79.95
Tabasco	14.44	39,450	7.1	9.3	53.6	87.7	11.6	0.48	0.84	0.70	0.67	0.73	68.07
Tamaulipas	6.54	49,150	4.1	9.5	35.1	87.2	12.2	0.45	0.85	0.70	0.70	0.75	156.96
Tlaxcala	6.01	40,301	3.7	9.3	48.4	92.8	7.1	0.425	0.845	0.685	0.671	0.730	44.63
Veracruz	7.8	32,445	3.4	8.2	61.8	88.7	10.8	0.534	0.834	0.648	0.638	0.701	76.26
Yucatán	15.29	48,879	2.1	8.8	40.8	89.1	10.7	0.462	0.837	0.685	0.703	0.739	111.14
Zacatecas	6.71	37,957	2.7	8.6	46.8	90.0	9.5	0.521	0.842	0.681	0.662	0.724	102.97

*SR, Suicide Rate; Income, Income per capita, Ue, unemployed; EL, Educational level, PP, Poor population, FH, Family households; Non-FH, Non-family household; GINI, GINI Index; HI, Health Index; EI, Education Index; InI, Income Index; HDI, Human Development Index; DR, Depression incidence rate*.

* National Institute of Statistics and Geography for year 2017;

** *National Council for the Evaluation of Social Development Policy for year 2018; + General Directorate of Epidemiology of the Ministry of Health in Mexico in the year 2018*.

In relation to the HDI of the 32 states of Mexican Republic, the medium HDI (score 0.741) was the most frequent, present in 41% of the states; while 22 and 13% correspond to high HDI and very high HDI, respectively; and 16% of the states have low HDI. The health index in general was medium (0.741), in contrast, the education and income indexes were low (0.692 and 0.696, respectively). The depression incidence rate was 111 cases per 100,000 inhabitants (Table 1).

The difference of the means in the study sample with low and high suicide rates, concerning the national mean, was 3.68, *t* = −22.8; *p* > 0.01, observing that only the health index showed a significant difference, *t* = −2.22; *p* > 0.05; the other study variables did not show significant differences (Table 2).

**Table 2:** Differences in study variables in the States of the Mexican Republic with low and high suicide rate.

	**T**	**g**	**Significance (bilateral)**	**Mean difference**	**SE**	**95% C.I**.	
						**Lower**	**Higher**
Suicide rate	−2.28	30	0.029	−3.68	1.61	−6.97	−0.39
Family households	0.58	30	0.562	0.94	1.6	−2.33	4.22
Non-family households	0.58	30	0.562	0.94	1.6	−2.33	4.22
Income index	−1.29	30	0.206	−0.029	0.023	−0.077	0.01
Education index	−0.46	30	0.647	−0.013	0.028	−0.071	0.045
Health index	−2.22	30	0.034	−0.012	0.005	−0.024	−0.001
Human development index	−1.03	30	0.312	−0.019	0.039	−0.057	0.018
Depression rate	−0.145	30	0.885	−5.74	39.5	−86.42	74.93

*SE, Standard Error*.

The results of the correlations showed that there is a significant and positive intensity relationship between the suicide rate and non-family households, with Pearson's correlation (*r* = 0.352; *p* < 0.001), that is, as they increase in non-family households, the suicide rate also increases. On the other hand, the suicide rate is significantly and negatively related to family households with Pearson (*r* = −0.350; *p* < 0.001), in other words, to the extent that family households increase, then, the rate of suicides decreases (Table 3).

**Table 3:** Correlation of study variables with the Suicide Rate in population aged 15 to 49 years old.

	**SR**	**I**	**Ue**	**EL**	**PP**	**FH**	**Non-FH**	**GINI**	**HI**	**EI**	**InI**	**HDI**	**DR**
SR	1	0.195	0.227	0.134	−0.099	−0.350^*^	0.352^*^	0.042	0.114	0.071	0.212	0.142	0.006
Income	0.195	1	0.344	0.888^**^	−0.897^**^	−0.567^**^	0.547^**^	−0.128	0.873^**^	0.795^**^	0.988^**^	0.943^**^	0.216
Ue	0.227	0.344	1	0.528^**^	−0.301	−0.197	0.178	−0.095	0.491^**^	0.458^**^	0.348	0.444^*^	0.073
EL	0.134	0.888^**^	0.528^**^	1	−0.835^**^	−0.542^**^	0.532^**^	−0.248	0.887^**^	0.880^**^	0.897^**^	0.952^**^	0.271
PP	−0.099	−0.897^**^	−0.301	−0.835^**^	1	0.537^**^	−0.530^**^	0.277	−0.854^**^	−0.610^**^	−0.932^**^	−0.826^**^	−0.456^**^
FH	−0.350^*^	−0.567^**^	−0.197	−0.542^**^	0.537^**^	1	−0.996^**^	0.033	−0.533^**^	−0.455^**^	−0.562^**^	−0.540^**^	−0.146
Non-FH	0.352^*^	0.547^**^	0.178	0.532^**^	−0.530^**^	−0.996^**^	1	−0.048	0.518^**^	0.447^*^	0.548^**^	0.528^**^	0.168
GINI	0.042	−0.128	−0.095	−0.248	0.277	0.033	−0.048	1	−0.226	−0.081	−0.225	−0.174	−0.205
HI	0.114	0.873^**^	0.491^**^	0.887^**^	−0.854^**^	−0.533^**^	0.518^**^	−0.226	1	0.769^**^	0.886^**^	0.899^**^	0.265
EI	0.071	0.795^**^	0.458^**^	0.880^**^	−0.610^**^	−0.455^**^	0.447^*^	−0.081	0.769^**^	1	0.767^**^	0.940^**^	0.103
InI	0.212	0.988^**^	0.348	0.897^**^	−0.932^**^	−0.562^**^	0.548^**^	−0.225	0.886^**^	0.767^**^	1	0.938^**^	0.280
HDI	0.142	0.943^**^	0.444^*^	0.952^**^	−0.826^**^	−0.540^**^	0.528^**^	−0.174	0.899^**^	0.940^**^	0.938^**^	1	0.214
DR	0.006	0.216	0.073	0.271	−0.456^**^	−0.146	0.168	−0.205	0.265	0.103	0.280	0.214	1

*SR, Suicide Rate; I, Income per capita; Ue, unemployed; EL, Educational level; PP, Poor population; FH, Family households; Non-FH, Non-Family household; GINI, GINI Index; HI, Health Index; EI, Education Index; InI, Income Index; HDI, Human Development Index; DR, Depression incidence rate*.

*** *p < 0.01*,

** *p < 0.05*,

* *p < 0.1*.

The explanatory-indicator variable with the highest relationship with the suicide rate is non-family households, which, in turn, correlated positively with the HDI, as well as HI, EI, and II. On the contrary, non-family households are only negatively associated with poverty (*r* = −0.530; *p* < 0.01); indeed, as non-family households increase, poverty decreases, but the rate of suicides increases (*r* = 0.352; *p* < 0.05) (Table 3).

Additionally, family households are positively associated with poverty, as family households increase, the percentage of poverty increases, but they are negatively associated with HI, EI, II, HDI, and especially with the suicide rate, that is, as family households increase, the indicators of human and social development decrease, in the same way, the suicide rate decreases (*r* = −0.350; *p* < 0.05) (Table 3).

On the other hand, those states of the country with low suicide rates were generally classified in the medium HDI category; whereas the high suicide rates were concentrated in those states with high HDI; even though, most of the country has suicide rates below the national average and a medium or low HDI. Moreover, a greater distribution of low suicide rates is also observed in the low education index, and medium health index. Regarding the incidence of depression and income, there is no predominant concentration. The most relevant result was the negative correlation between family household rates and the suicide rate.

The MLR analysis showed that the Income Index, Depression Incidence and HDI, did not predict the suicide rate. Unlike, the variable family household which significantly predicted (*p* = 0.04) a decrease in the suicide rate, the coefficient indicates that, for each percentage increase in family homes, a 0.4% decrease in the suicide rate can be expected (Table 4).

**Table 4:** Multiple linear regression: protective contribution of Family households on the suicide rate.

**Dependent variable: suicide rate**	
Family households	−0.395^*^ (0.19)
Income index	122.04 (487.60)
Depression incidence	−0.002 (0.008)
HDI	−5.81 (19.21)
Constant	43.69^**^ (16.94)
*R*^2^	0.124

* *p < 0.05*,

** *p < 0.01*.

*The estimated regression coefficients appear first, followed by the standard deviations of the parameters calculated from of the variance using the stepwise method (step by step). R^2^ is the coefficient of determination*.

## Discussion

The purpose of this study was to evaluate the probability of suicide rates in Mexico in the 15–49 years old population as a function of the depression incidence rate, the Human Development Index, other variables of social development as Health, Education, and Income Indexes as well as the household structure.

The findings of the present study revealed that depression incidence rate was not positively associated with suicide rate in young population between 15 and 49 years of age despite depression is a known risk factor for suicide. According to the results of the investigation, there is a direct positive association between high HDI and depression, but not with low or medium HDI. Moreover, it is possible that in the states with low or medium HDI, there is less access to mental health services; consequently, there is less record of cases of depression; naturally, deaths, including those by suicide, are registered, and reported in vital statistics. Because of this, the probability to commit suicide could be similar between the states with low and high HDI. In spite of the fact that depression is highly prevalent in adolescents in LMICs and that it is the strongest predictor of suicide, as recently suggested, in these countries more research is still needed to understand this association being the effectiveness of depression and suicide prevention intervention “limited at best” (22).

Although, the human development *per se* does not promote suicidal behavior, but possibly the social and individual effect of the change in social relationships, rhythm of life or stress could be factors related to suicidal behavior (23).

However, within there are factors such as depression and loneliness have been described as significant predictors of suicide risk, above all in countries with high HDI (24). The suicide rate is higher in high HDI countries, also in those countries, a higher incidence of people who feel alone has been reported, an incidence that can reach up to 40% (25). This is in line with various studies that have provided evidence of HDI and suicide, for example, worldwide 52% of suicides have occurred in countries with high HDI (9). In these countries, loneliness has been found to increase the risk of suicidal behavior (26). The phenomenon of loneliness is defined as a discrepancy between current social relationships and individual preference; the discrepancy leads to a negative experience of feeling alone, and dysphoria of feeling socially isolated, even when the person is accompanied by family and friends (27). In this perspective, loneliness is an independent phenomenon of depression with well-defined dimensions (28).

A longitudinal study carried out in countries with high HDI found that high levels of feelings of loneliness are significantly correlated with suicidal ideation in adult population (29). This is in agreement with cross-sectional large sample in young-and-middle age adults in Europe studies that have documented that higher isolation being female and with chronic health conditions are related to 12-month suicidal ideation (30). In this sense, the family household variable predict reduction of suicide rate and positive correlation between the suicide rate and non-family households, in the present research, is possibly mediated by the lack of perception of social support, understood as the level of satisfaction that an individual feels regarding the comprehension and support of the environment in which they develop (31). This is an aspect that should be analyzed in future studies in Mexico Latin America and other countries to understand the suicide phenomenon given the fact that the subjective feeling of loneliness that accompanies social isolation have a transcultural dimension (32) that future research should address

It has also been found that having low educational level, being underemployed, living in urban areas, being a woman, not being married, and being separated, increases the risk of suicidal behavior (33). This finding reported in HMICs, and others factors such as unemployment, financial stress, and associated family instability can lead to poverty and influence suicidal behavior (34). Although a recent systematic review of studies in LMICs revealed a positive association between poverty and suicide, as the authors emphasize, this trend of knowledge is more consistent at an individual level because at a country level, data is sparse to reach unanimous conclusions (35).

The states of the Mexican Republic with the highest prevalence of non-family households had a positive association with the suicide rate. Based on the result of this study, it is possible to assume that, as the HDI increases, there is a greater possibility of living alone and having suicidal behavior; furthermore, the non-family household variable was negatively associated with poverty and a low index of income and educational level. In a broader context, the results highlight that both depression, loneliness, and variables related to the HDI affect the suicide rates.

These findings contribute to a more detailed view of the ways in which the suicidal behavior can occur. This is a problem that the Mexican health system must urgently confront, on one hand, it must consider that the country is in a stage of social transition and economic uncertainty that has an impact on suicide rates, and on the other hand, that there are mental health care needs in the population. Therefore access to mental health services must be improved, this is particularly important in the male young population since it is pointed out that the lack of focus or direction to the future has been related to an increased risk of suicidal behavior (36).

All the above represent for the Mexican health system a significant challenge. The analysis of the needs of health services shows that, even though there is high prevalence of mental disorders as depression, there is scarcity and inequality in the distribution of mental health resources. There is no model of care based on primary care. There is also a reduction in beds in Psychiatric Hospitals or General Hospitals for the care of patients with severe mental disorders, as would be the case for patients with suicide risk (37).

The authors want to highlight that the understanding of social determinants is essential to illustrate the potential of primary prevention. In suicide prevention, there are two perspectives that must be addressed, one of them is the prevalence of depression as a precedent to suicidal behavior (and suicide), this is a growing problem that the health system is responsible for and has not adequately tackled; the other one is the way various factors and social problems influence human development and individual adaptation associated to suicide; which emphasizes the need for the health system to connect with other social sectors to generate true support networks and promote integral human development, thus achieving the health system's objective while helping to reach a sustainable development.

## Data Availability Statement

Publicly available datasets were analysed in this study. These can be found here: https://datos.gob.mx/busca/dataset/proyecciones-de-la-poblacion-de-mexico-y-de-las-entidades-federativas-2016-2050; www.epidemiologia.salud.gob.mx/anuario/html/anuarios.html; https://www.inegi.org.mx/app/tabulados/interactivos/?px=Mortalidad_01&bd=Mortalidad; https://www.inegi.org.mx/app/tabulados/interactivos/default?px=Hogares_10&bd=Hogare; https://www.inegi.org.mx/app/tabulados/interactivos/default?px=Educacion_05&bd=Educacion; https://www.coneval.org.mx/Medicion/Paginas/PobrezaInicio.aspx.

## Author Contributions

HC-R contributed with original conception, design, data collection, and analysis. MM-C contributed data analysis, manuscript writing, and critical review. LD-C contributed to the design of the work, data analysis, writing of the manuscript, and critical revision. All authors contributed to the article and approved the submitted version.

## Conflict of Interest

The authors declare that the research was conducted in the absence of any commercial or financial relationships that could be construed as a potential conflict of interest.
